# The Role of Dietary Inflammatory Index in Cardiovascular Disease, Metabolic Syndrome and Mortality

**DOI:** 10.3390/ijms17081265

**Published:** 2016-08-03

**Authors:** Miguel Ruiz-Canela, Maira Bes-Rastrollo, Miguel A. Martínez-González

**Affiliations:** 1 Department of Preventive Medicine and Public Health, University of Navarra, Pamplona 31008, Spain; mbes@unav.es (M.B.-R.); mamartinez@unav.es (M.A.M.-G.); 2 IDISNA (Navarra Health Research Institute), Pamplona 31008, Spain; 3 Centro de Investigación Biomédica en Red Fisiopatología de la Obesidad y Nutrición (CIBERobn), Instituto de Salud Carlos III, Madrid 28029, Spain

**Keywords:** dietary inflammatory index, inflammation, cardiovascular disease, type-2 diabetes, metabolic syndrome, mortality

## Abstract

Inflammation is an underlying pathophysiological process in chronic diseases, such as obesity, type 2 diabetes mellitus and cardiovascular disease. In fact, a number of systematic reviews have shown the association between inflammatory biomarkers, such as CRP, IL-1β, IL-6, TNF-α, IL-4, or IL-10, and cardio-metabolic diseases. Diet is one of the main lifestyle-related factors which modulates the inflammatory process. Different individual foods and dietary patterns can have a beneficial health effect associated with their anti-inflammatory properties. The dietary inflammatory index (DII) was recently developed to estimate the inflammatory potential of overall diet. The aim of this review is to examine the findings of recent papers that have investigated the association between the DII, cardio-metabolic risk factors and cardiovascular disease. The relevance of the DII score in the association between inflammation and cardio-metabolic diseases is critically appraised, as well as its role in the context of healthy dietary patterns. We conclude that the DII score seems to be a useful tool to appraise the inflammatory capacity of the diet and to better understand the relationships between diet, inflammation, and cardio-metabolic diseases.

## 1. Introduction

Inflammation is now widely believed to be a cause of atherosclerosis [1,2]. Moreover, inflammation is an underlying pathophysiological mechanism in many other chronic diseases, including obesity [3] and type 2 diabetes mellitus [4], in addition to cardiovascular disease (CVD) [5]. Multiple factors contribute to this inflammatory process, including age, sex, physical activity, smoking, the use of certain medications, and diet [6]. Therefore, lifestyle and diet have a significant impact on health which is based, at least in part, on its association with inflammation [7,8].

Diet is a major determinant of inflammation and a number of markers can be used to assess the inflammation in human nutrition studies [9,10]. Pro-inflammatory biomarkers, such as tumor necrosis factor-α (TNF-α), C-reactive protein (CRP), or cell adhesion molecules have been used as indices of the effect of dietary patterns on low-grade inflammatory status [11,12,13,14,15]. In fact, the inverse association of a Mediterranean-style diet with major chronic disease is partially attributed to the anti-inflammatory properties of some of their foods such as fruits, extra-virgin olive oil, red wine, or nuts [16,17], and some of their bioactive components, such as polyphenols [18]. In contrast, certain components of diet, such as red meat and processed foods, are considered to be pro-inflammatory stimulants [19,20].

Currently, there is an increasing interest in the anti-inflammatory properties of the food patterns for the prevention of cardio-metabolic and other chronic diseases [21]. The characterization of diet according to its inflammatory properties can be useful to investigate the link between diet and CVD. The dietary inflammatory index (DII) was developed to estimate the inflammatory potential of the overall food pattern [22]. The aim of this review is to examine the findings of all the studies that have investigated the association between the DII score, cardio-metabolic risk factors, and CVD.

## 2. Methods

In May 2016, we conducted a search in PubMed and afterwards a hand-search of all references included in the identified articles. The search strategy included the terms “dietary inflammatory index”, “cardiovascular disease”, “metabolic syndrome”, and “death” or “mortality”. The eligibility criteria included any observational epidemiologic study, either cross-sectional or prospective, which had used the modified dietary inflammatory index designed in 2013 by Shivappa et al. [22], and the estimation of a multivariable-adjusted relative risk (and 95% confidence interval) comparing quantiles of the DII score with respect to the risk of CVD, metabolic syndrome or death, either as a primary or as a secondary outcome.

## 3. The Dietary Inflammatory Index (DII) Score

The DII is a score used to determine the overall inflammatory potential of diet. This index is based on 1943 articles, published from 1950 and 2010, reporting the effect of 45 dietary parameters on six inflammatory biomarkers [22]. Each one of these dietary parameters received a positive score (+1) if its effect was pro-inflammatory (significantly increased IL-1β, IL-6, TNF-α, or CRP, or decreased IL-4 or IL-10), a negative score (−1) if its effect was anti-inflammatory and 0 if no significant change in biomarkers associated to that dietary parameter was found. Individuals’ intake of each food parameter was subtracted from a world global standard database and then divided by the world standard deviation for each food parameter. These values were converted to a percentile score, each percentile was doubled, and then 1 was subtracted to achieve a symmetrical distribution (from −1 to +1 and centered on 0). Afterwards, each one of these values was multiplied by the overall food parameter specific inflammatory score. Finally the sum of all the food parameter-specific DII scores provided the overall DII score for each individual. Thus, positive DII scores represent a pro-inflammatory diet and negative DII scores represent an anti-inflammatory diet. For example, this score can have values ranging from 7.98 to −8.87 in different scenarios in the design and developing of the DII score [22].

Several studies have shown the association between the DII score and inflammatory biomarkers, including the rate of telomere shortening [20,21,22,23]. In a longitudinal analysis of 559 healthy participants, higher DII scores were associated with values of high-sensitivity(hs)-CRP greater than 3 mg/L [23]. In a cross-sectional analysis with 2524 healthy participants, the DII score was positively associated with IL-6 (>1.6 pg/mL) and homocysteine (>15 μmol/L), although no significant association was found with high-sensitivity CRP and fibrinogen [24]. The DII score was also associated with IL-6, TNF-α, and hs-CRP among 2567 postmenopausal women [25]. In the PREDIMED-Navarra study, a greater DII score was associated with almost a two-fold higher risk of telomere shortening compared with the anti-inflammatory values of the DII score during a five-year follow-up period [26].

## 4. Cardiovascular Disease and DII Score

We found four studies assessing the association between the DII score and incident CVD [27,28,29,30], and another study with previous CVD [31]. Table 1 shows a summary of these studies with the relative risk for the main outcome in the most fully-adjusted model. Results were consistent showing in four of the five studies a direct association, meaning that a greater pro-inflammatory diet is related to a higher risk of CVD. There was some heterogeneity regarding several characteristics of the studies, as detailed below in Table 1. There was a variety regarding the number of food parameters used to calculate the DII score as well as the age of participants, duration of follow-up, and definition of cases. However, the relative risk estimates were consistent across the studies.

The Geelong osteoporosis study was a population-based study including 1363 men with an age of 18 or older. Among them, 76 had CVD resulting in hospitalization over a period of five years of follow-up [27]. Participants were dichotomized according to the anti-inflammatory or pro-inflammatory capacity of diet (as captured by the DII score). CVD cases were collected retrospectively from medical records in 2011. Compared with participants following an anti-inflammatory diet pattern, the adjusted OR (95% confidence interval) for CVD was 2.00 (1.01–3.96) for those with positive values of the DII score (pro-inflammatory diet). The biggest impact was found when including only those events that occurred during the first three years of follow-up.

The PREDIMED study assessed 7216 men (55 to 80 years) and women (60 to 80 years) at high risk of CVD [28]. The number of CVD events (MI, stroke or cardiovascular death) was 277 for a median follow-up of over 4.5 years. Using the lowest quartile of the DII score as the reference, the adjusted hazard ratio (95% confidence interval) for CVD was 1.73 (1.15–2.60) for those participants exposed to the highest quartile (most pro-inflammatory diet at baseline). A stronger association was found when cases occurring during the first year of follow-up were excluded from the analysis.

The SUN cohort assessed the association between the DII score and incident CVD in 18,794 middle-age participants with a median follow-up of 8.9 years [29]. The number of new-onset CVD cases was 117 (MI, stroke and CVD death). The adjusted HR for participants in the highest (most pro-inflammatory) vs. the lowest quartile of the DII score was 2.03 (95% CI 1.06–3.88). The estimated risk was higher after excluding those with CVD events occurring after five years of follow-up.

**Figure 1:**
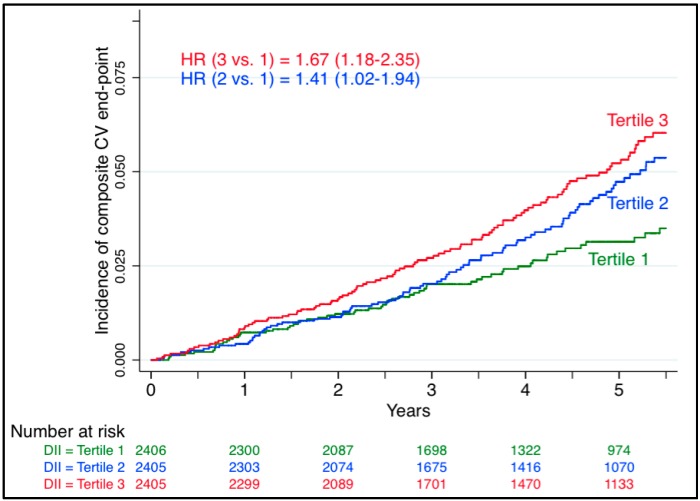
Cumulative incidence of CVD in the PREDIMED study [28] according to tertiles of the DII score.

**Figure 2:**
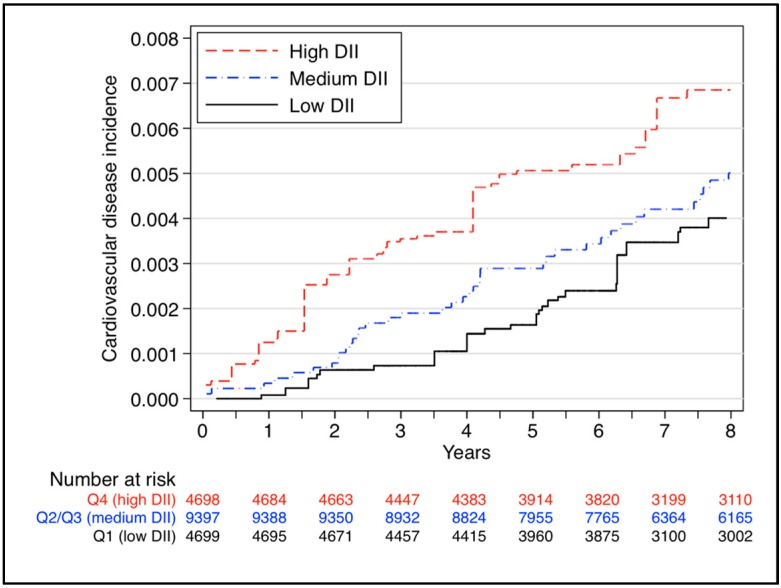
Cumulative incidence of CVD in the the SUN cohort [29] according to quartiles of the DII score (merging the two intermediate quartiles to build a medium category). The data are adjusted for sex, age, hypertension, dyslipidemia, diabetes, smoking, family history of premature CVD, and total energy intake.

Figure 1 and Figure 2 show the cumulative incidence of CVD in the PREDIMED study [28] and the SUN cohort [29] according to the tertiles and quartiles of the DII score, respectively. Both curves indicate that the highest values of the DII score (the most pro-inflammatory diet in red color) had a higher incidence of CVD compared with lower values during the follow-up of the studies.

The SU.VI.MAX study included 7743 women (aged 35–60 years) and men (aged 45–60 years) [30]. A total of 292 CVD cases (MI, stroke and angina pectoris, or revascularization intervention) were included during a mean follow-up of 11.4 years. In this study no statistically significant association between the DII score and the composite CVD outcome was observed (Table 1). However, a significant association was found for MI when comparing the highest vs. the lowest quartile of the DII score and this association was slightly higher when excluding MI cases occurring during the first two years of follow-up.

The NHANES study explored the association between the DII score and a composite outcome of 1734 self-reported previously diagnosed CVD cases (including congestive heart failure, coronary heart disease, angina, heart attack, and stroke) [31]. In the analyses for each disease, significant direct associations were found for congestive heart failure, heart attack, stroke, and hypertension, but not for coronary heart disease and angina pectoris. In the stratified analysis by sex, a significant association was found in women, but not men (*p* for interaction < 0.01).

## 5. Metabolic Syndrome and the DII Score

Regarding the association between DII score and metabolic syndrome (MetSyn), we found four studies (Table 2). No significant association was found in two of them with a cross-sectional design [32,33] and in a prospective study [34]. On the contrary, a significant association was found in a cohort study using a greater number of food parameters to obtain the DII score [35].

No association was found between the DII score and MetSyn in a cross-sectional analysis with 3862 participants from the Polish-Norwegian Study [32]. Participants in this study were men and women between 45 and 64 years and the prevalence of MetSyn was 30%. The HR for metabolic syndrome was 0.96 (95% CI: 0.77–1.19), when comparing quartile 4 vs. 1 of the DII score. Similarly, no significant association was found for the individual components of the MetSyn except for an inverse association between HDL cholesterol and the DII score (Q4 vs. Q1: OR = 0.62; 95% CI: 0.48–0.80). On the contrary, in the stratified analysis by sex, an inverse association was found between DII score and MetSyn among women [32]. This association might be due to reverse causation in this cross-sectional study.

A lack of association between the DII score and MetSyn was also found in a cross-sectional study with 464 participants from the Buffalo Cardio-Metabolic Occupational Police Stress study [33]. The mean age of these participants was 42 years and the prevalence of MetSyn was 28%. There was also no association between the DII score and each of the individual components of the MetSyn except for the glucose intolerance component (OR = 2.03; 95% CI: 1.08–3.82 for quartile 4 vs. 1). Again, the cross-sectional design and the small sample size might explain this lack of association.

Pimenta et al. studied the association between different dietary indexes and the MetSyn in participants from the SUN cohort [34]. The number of participants with incident MetSyn was 346 among 6851 participants followed during a mean of 8.3 years. They did not find a statistical significant association between the DII score and MetSyn after adjusting for other potential confounders.

In the SU.VI.MAX study, 3726 participants (mean age 50 years) were included to assess the association between the DII score and the MetSyn [35]. After a mean follow-up of 12.4 years, 524 (14%) participants developed MetSyn. The odds of MetSyn was 39% higher among those with the greatest pro-inflammatory diet (quartile 4 of the DII score) compared with those with the most anti-inflammatory diet (quartile 1). A higher DII score was also significantly associated with higher diastolic and systolic blood pressure, higher triglycerides levels and lower HDL-cholesterol.

Finally, in a cross-sectional analysis with 7236 participants from the PREDIMED study, a direct association was found between higher (pro-inflammatory) levels of the DII score and waist circumference which is one of the components of the MetSyn [36].

## 6. Mortality and the DII Score

Table 3 shows five studies studying the association between the DII score and all-cause mortality [37,38,39,40,41]. In all studies except one, a higher risk of all-cause death was found among those participants with the highest pro-inflammatory values of the DII score. Using the third National Health and Nutrition Examination Survey (NHANES III), Shivappa et al. found a direct association between a higher pro-inflammatory diet and mortality in participants with ages above 19 years [37]. In the same study, significant associations were also found between the DII score and all-cancer mortality (HR = 1.46; 95% CI: 1.10–1.96), digestive-tract cancer mortality (HR = 2.10; 95% CI: 1.15–3.84), and CVD mortality (HR = 1.46; 95% CI: 1.18, 1.81). Similar results were found in another analysis with participants from the NHANES III after stratifying by the diabetic status of participants [38]. In this case, the highest risk of all-cause mortality was found among participants with prediabetes (serum HbA1c between 5.7% and 6.4%), as it is shown in Table 3.

The Iowa Women’s Health study found a more modest association between the DII score and total mortality [39]. This analysis included 37,525 women aged 55–69 at baseline and with a mean follow-up of 20.7 years. There were also significant and direct associations between a higher pro-inflammatory level in the DII score and cancer mortality (for all cancers and for digestive cancers), CVD mortality, and chronic obstructive pulmonary disease related mortality.

Interestingly, the SU.VI.MAX study did not find any significant association between the DII score and all-cause mortality [40]. During a median follow-up of 12.4 years, 207 out of 8089 participants died. The DII score was positively associated with CVD mortality (HR = 1.53; 95% CI: 1.01–2.32) and cancer mortality (HR = 1.83; 95% CI: 1.12, 2.99) but no association was found with all-causes mortality (HR = 1.41; 95% CI: 0.97–2.04). However, in the stratified analysis by intervention group, a statistical association was found between the DII score and all-cause mortality in the placebo group but not in the antioxidant-supplemented group (Table 3).

Finally, the Swedish Mammography Cohort including a large sample size (*n* = 33,747) found a direct association of the DII score with all-cause mortality as well as with CVD mortality (HR = 1.35; 95% CI: 1.01–1.81) when comparing extreme quintiles [41]. However, no significant association was found for cancer mortality or digestive-cancer mortality.

## 7. The Relevance of the DII Score in the Association between Inflammation and Cardio-Metabolic Diseases

There is a clear association between the inflammatory biomarkers used to calculate the DII score (CRP, IL-1β, IL-6, TNF-α, IL-4, and IL-10) [22] and cardio-metabolic diseases such as obesity, diabetes, or CVD. Specifically, higher levels of CRP were correlated with BMI, waist circumference, or waist-to-hip ratio in a meta-analysis with 53 cross-sectional studies [42]. A cohort study with women also found this association [43]. Though two Mendelian randomization studies have suggested that probably adiposity causes inflammation but not vice versa [44,45], the bidirectional association is possible and a pro-inflammatory dietary exposure can precede the development of obesity [46,47]. Recently, a review on hypothalamic micro inflammation suggested a potential mechanism link between a pro-inflammatory diet and MetSyn [48]. Regarding diabetes, a meta-analysis with 16 studies found an association between hs-CRP and incident diabetes [49] although a nested case-control study in the European Prospective Investigation of Cancer (EPIC)-Norfolk cohort suggested that this association might be confounded by central adiposity [49]. With respect to CVD, higher hs-CRP was independently associated with carotid intima-media thickness [50]. An individual participant meta-analysis from 54 long-term prospective studies reported an association between hs-CRP concentration and the risk of coronary heart disease, stroke and both vascular and non-vascular mortality [51]. However, this association is not completely admitted because a Mendelian association meta-analysis of individual participant data did not confirm it [52]. However, Mendelian randomization studies are not free of biases and may have some important limitations mainly derived from their underlying assumptions that are not always valid [53].

Although CRP is clinically relevant for risk prediction, it is considered a surrogate biomarker of upstream cytokines, such as IL-6 and IL-1β [54]. A systematic review found that MetSyn is associated with elevated concentrations of IL-6 and TNF-α and with decreased levels of IL-10, an anti-inflammatory biomarker used in the calculation of the DII score [55]. However, this association between IL-10 and diabetes is still less studied and there are no available prospective studies to support this association [56]. The Leiden 85-Plus Study showed a cross-sectional association between a low IL-10 production capacity and both MetSyn and type-2 diabetes [57]. Regarding coronary disease, a meta-analysis of 29 prospective studies found that higher baseline levels of pro-inflammatory cytokines, including IL-6 and TNF-α, were associated with a greater risk of non-fatal myocardial infarction or CVD death [58]. Two mendelian randomization studies have suggested IL-6 receptors to have a causal role on coronary heart disease [59,60]. Although anti-inflammatory IL-10 was traditionally considered protective against atherosclerosis [61], a prospective study found that circulating IL-10 concentrations at baseline were positively associated with nonfatal myocardial infarction, stroke, or CVD death [62]. Similarly, a growing body of evidence suggests that IL-4, previously considered an anti-inflammatory cytokine, could be involved in the initiation and progression of atherosclerosis [63].

In general, all this scientific evidence is highly relevant to explain the association between dietary exposures and CVD, MetSyn, and mortality, which can be mediated by a pro-inflammatory exposure through the food pattern. As previously mentioned, the DII score seems to be able to capture this exposure because it is based on the role that 45 foods and dietary constituents have on six well-acknowledged inflammatory biomarkers [22]. Among these biomarkers, those with a pro-inflammatory effect (hs-CRP, IL-1β, IL-6 and TNF-α) are associated with cardiometabolic risk factors and CVD, as we have explained above. There is still a limited knowledge regarding the role of IL-10 and IL-4 and this is probably a limitation of the DII score. The construction of the DII score is based on scientific knowledge available until 2010 and thus, an update will be needed to refine the inflammatory capacity of these cytokines as well as other new inflammatory markers.

## 8. The DII Score in the Context of Healthy Dietary Patterns

The use of dietary patterns is one of the best approaches to understand the relationship between diet and disease [64]. Dietary patterns are used to assess the general quality of a diet and takes into account the potential synergies between different foods [65]. One method for defining dietary patterns is the construction of dietary indices according to some specific dietary recommendations. For example, the Healthy Eating Index (HEI) followed the Dietary Guidelines for Americans 2005 [66] and it has been adapted according to changes in the dietary recommendations [67,68]. Other indices are more focused on some specific health benefit such as the Dietary Approaches to Stop Hypertension (DASH) diet [69], and other indices try to assess the level of adherence to traditional dietary patterns, like the Mediterranean diet (MedDiet) [70] or the vegetarian dietary pattern. Both the DASH and traditional MedDiet are the most well-studied dietary patterns [71].

All of these healthy dietary patterns share the protective effect against the most prevalent diseases such as cancer, cardiovascular diseases or diabetes. A meta-analysis of cohort studies found that the HEI, AHEI, and the DASH score were associated with a risk reduction for all-cause mortality, CVD, cancer, and type-2 diabetes [72]. The protective role of the MedDiet against these health outcomes has also been shown consistently in several systematic reviews [73,74]. Two systematic reviews [75,76] concluded that both the MedDiet and the DASH diet are effective for weight loss. The large and long-term PREDIMED randomized trial (www.predimed.es) recently also reported benefits for the Mediterranean diet for the prevention of age-related weight gain [77]. According to the 2015 Dietary Guidelines Advisory Committee (http://health.gov/dietaryguidelines/2015-scientific-report/) those dietary patterns associated with a decreased risk of CVD are characterized by a lower consumption of red and processed meat, lower intakes of sugar-sweetened foods and beverages and refined grains as well as higher consumption of fruits, vegetables, whole grains, low-fat dairies, and seafood [78]. In fact, several meta-analyses have shown the link between these individual foods and better cardiometabolic outcomes [71].

The DII score is inversely associated with healthy scores including the AHEI, HEI-2010, and DASH [79]. This is consistent with the fact that lower hs-CRP concentrations are associated with a higher intake of fruits and vegetables [80,81], legumes [82], nuts [83], and dietary fiber [84]. Western-style diets are positively associated with hs-CRP levels, whereas healthy diets are inversely associated with it [85,86]. A systematic review showed that higher adherence to the Mediterranean diet was associated with decreased levels of inflammatory biomarkers, including hs-CRP, interleukin-6, and intracellular adhesion molecule-1 [14]. Similarly, olive oil (a distinctive characteristic of the MedDiet) specially in its extra-virgin variety may also exert a powerful anti-inflammatory effect [87].

The DII score, in contrast to other dietary patterns, is focused on a pathophysiological process, i.e., how a number of foods modulate several markers involved in inflammation. In fact, the correlation with healthy indexes was not perfect [79] and, thus, the DII score probably accounts for other sources of variability related to inflammation and provides additional information beyond that provided by other dietary patterns. This makes very promising the construction of dietary patterns based on specific biological pathways (i.e., inflammation) and other alternative inflammatory indexes have been developed [88,89]. The strength of the DII score is the high number of studies showing an inverse association with cardiovascular-related diseases, as well as other chronic diseases, such as cancer [90,91,92,93].

## 9. Conclusions

Several studies have shown the important role of inflammation as a mechanism involved in the pathophysiological process of many chronic diseases. In this context, diet is a modifiable factor which is likely to exert a powerful pro-inflammatory or anti-inflammatory effect. The DII score seems to be a useful tool to appraise the inflammatory capacity of diet. A number of studies have consistently shown a direct association between the DII score and a higher risk of CVD, MetSyn, and overall mortality. The use of this score can be a recommended approach to understand the relationships between diet, inflammation, and cardio-metabolic diseases.

## Figures and Tables

**Table 1:** Association between the DII score and the incidence of cardiovascular disease.

Study Name	Design	# Food Parameters	Follow-up ^1^ (Years)	N Total (CVD Cases)	Groups	Adjusted Relative Risk (95% CI)	Covariables
GOS ^2^ [27]	Cohort	22 using FFQ	5	1363 (76)	Negative DII (ref) vs. Positive DII	OR = 2.00 (1.01–3.96)	Family history of CVD, blood pressure, sedentary, diabetes, smoking, waist circumference, age, total energy intake
PREDIMED ^3^ [28]	Cohort	32 using FFQ	4.7	7216 (277)	Quartile 1 (ref) vs. Quartile 4	HR = 1.73 (1.15–2.60)	Age, sex, overweight/obesity, waist-to-height ratio, total energy intake, smoking status, diabetes, hypertension, dyslipidemia, family history of premature cardiovascular disease, physical activity, educational level, intervention group, center
SUN ^4^ [29]	Cohort	28 using FFQ	8.9	18,794 (117)	Quartile 1 (ref) vs. Quartile 4	HR = 2.03 (1.06–3.88)	Age, sex, hypertension, dyslipidaemia, diabetes, smoking status, family history of cardiovascular disease, total energy intake, physical activity, body mass index, educational level, other cardiovascular diseases, special diet at baseline, snacking, average time sitting, average time spent watching television
SU.VI.MAX ^5^ [30]	Cohort	36 using 24-h dietary records	11.4	7743 (292)	Quartile 1 (ref) vs. Quartile 4	HR = 1.16 (0.79–1.69)	Sex, energy intake, supplementation group, number of 24-h records, education level, marital status, smoking status, physical activity, body mass index
			11.4	7602 (93)	Quartile 1 (ref) vs. Quartile 4	HR = 2.26 * (1.08–4.71)	
NHANES ^6^ [31]	Cross-sectional	27 using 24-h dietary records	NA	15,693 (1734)	Quartile 1 (ref) vs. Quartile 4	OR = 1.30 (1.06–1.58)	Family member smoking status, personal smoking status, age, body mass index

^1^ Mean or median except the GOS study; ^2^ GOS: Geelong Osteoporosis study; ^3^ PREDIMED: Prevention with Mediterranean Diet (PREvención con DIeta MEDiterránea); ^4^ SUN: University of Navarra Follow-up (Seguimiento Universidad de Navarra); ^5^ SU.VI.MAX: Antioxidant Vitamins and Minerals Supplementation (SUpplémentation en VItamines et Minéraux AntioXydants); ^6^ NHANES: National Health and Nutrition Examination Survey III follow-up; * HR for myocardial infarction in the stratified analysis; **#**: number; ref: reference.

**Table 2:** Association between the DII score and the metabolic syndrome ^1^.

Study Name	Design	# Food Parameters	Follow-up (Years)	N Total (Cases)	Groups	Adjusted Relative Risk (95% CI)	Covariables
PONS ^2^ [32]	Cross-sectional	22 using FFQ	NA	3862 (1159)	Quartile 1 (ref) vs. Quartile 4	OR = 0.96 (0.77–1.19)	Body mass index, age
BCOPS ^3^ [33]	Cross-sectional	Not reported	NA	464 (125)	Quartile 1 (ref) vs. Quartile 4	OR = 0.87 (0.46–1.63)	Age, sex
SUN ^4^ [34]	Cohort	28 using FFQ	8.3	6851 (346)	Quintile 1 (ref) vs. Quintile 5	HR * = 0.86 (0.60–1.23)	Age, sex, smoking, alcohol consumption, snacking between main meals, use of special diets, television watching, physical activity, changes in weight over the last 5 years prior, body mass index
SU.VI.MAX ^5^ [35]	Cohort	36 using 24-h dietary records	12.4	3726 (524)	Quartile 1 (ref) vs. Quartile 4	HR = 1.39 (1.01–1.92)	Age, sex, supplementation group, number of 24 h records, energy intake, education level, smoking status, physical activity, body mass index

^1^ MetSyn was defined as the presence of at least three of these components: Abdominal obesity; high blood pressure; low HDL cholesterol; high triglycerides and high glucose level; ^2^ PONS: Polish-Norwegian Study; ^3^ BCOPS: Buffalo Cardio-Metabolic Occupational Police Stress; ^4^ SUN: University of Navarra Follow-up (Seguimiento Universidad de Navarra); ^5^ SU.VI.MAX: Antioxidant Vitamins and Minerals Supplementation (SUpplémentation en VItamines et Minéraux AntioXydants); **#**: Number; ref: reference; * Data provided by the authors.

**Table 3:** Association between the DII score and all-cause mortality.

Study Name	Design	# Food Parameters	Follow-up (Years)	N Total (Cases)	Groups	Adjusted Relative Risk (95% CI)	Covariables
NHANES ^1^ III [37]	Cohort	27 using 24-h dietary records	13.5	12,438 (2795)	Tertile 1 (ref) vs. Tertile 3	HR = 1.34 (1.19, 1.51)	Age, sex, race, diabetes status, hypertension, physical activity, BMI, poverty index, smoking
NHANES ^1^ III [38]	Cohort	27 using 24-h dietary records	NA	2681 (896)	Tertile 1 (ref) vs. Tertile 3	HR = 1.39 (1.13, 1.72)	Age, sex, race, HbA1C, current smoking, physical activity, body mass index, systolic blood pressure
Iowa Women’s Health study [39]	Cohort	37 using FFQ	20.7	37,525 (17,793)	Quartile 1 (ref) vs. Quartile 4	HR = 1.08 (1.03–1.13)	Age, body mass index, smoking status, pack-years of Smoking, hormone replacement therapy use, education, diabetes, hypertension, heart disease, cancer, total energy intake
SU.VI.MAX ^2^ [40]	Cohort	36 using 24-h dietary records	1.24	8089 (207)	Tertile 1 (ref) vs. Tertile 3	HR * = 2.10 (1.15–3.84)	Age, sex, intervention group, number of 24-hour dietary records, body mass index, physical activity, smoking status, educational level, family history of cancer in first-degree relatives, family history of CVD in first-degree relatives, energy intake without alcohol, and alcohol intake
						HR ** = 1.09 (0.67–1.77)	
Swedish Mammography Cohort [41]	Cohort	27 using FFQ	15	33,747 (7095)	Quintile 1 (ref) vs. Quintile 5	HR = 1.41 (1.21–1.64)	Age, energy intake, body mass index, education, smoking status, physical activity, alcohol intake

^1^ NHANES: National Health and Nutrition Examination Survey III follow-up; ^2^ SU.VI.MAX: Antioxidant Vitamins and Minerals Supplementation (SUpplémentation en VItamines et Minéraux AntioXydants); **#**: number; ref: reference; * HR for the placebo group; ** HR for the antioxidant supplementation group.

## Abbreviations

CRP	C-Reactive Protein
DII	Dietary Inflammatory Index
FFQ	Food Frequency Questionnaire
HR	Hazard Ratio
IL	Interleukin
MedDiet	Mediterranean Diet
MetSyn	Metabolic Syndrome
OR	Odds Ratio
TNF-α	Tumor Necrosis Factor-α
